# Crystal structure of poly[tetra-μ_2_-cyanido-1:2κ^8^ *N*:*C*-bis­(dimethyl sulfoxide-1κ*O*)diargentate(I)iron(II)]

**DOI:** 10.1107/S2056989017001049

**Published:** 2017-01-27

**Authors:** Olesia I. Kucheriv, Dina D. Naumova, Inna I. Tokmenko, Ruslan A. Polunin, Kateryna V. Terebilenko

**Affiliations:** a Department of Chemistry, Taras Shevchenko National University of Kyiv, Volodymyrska St. 64, Kyiv 01601, Ukraine; b National O.O. Bogomoletz Medical University, 13 T. Shevchenko Blvd., Kyiv, Ukraine; c L. V. Pisarzhevskii Institute of Physical Chemistry, National Academy of Sciences of Ukraine, Prospekt Nauky 31, Kyiv 01601, Ukraine

**Keywords:** crystal structure, Fe^II^ complex, Hofmann clathrate analogue, di­cyanido­argentate, metal–organic framework

## Abstract

In the title polymeric complex, [Fe{OS(CH_3_)_2_}_2_{Ag(CN)_2_}_2_], the Fe^II^ cation is located at an inversion centre and is coordinated by four cyanide (CN^−^) anions and two dimethyl sulfoxide mol­ecules in a slightly compressed N_4_O_2_ octa­hedral geometry, the Ag^I^ cation is C-coordinated by two CN^−^ anions in a nearly linear geometry. The CN^−^ anions bridge the Fe^II^ and Ag^I^ cations to form a two-dimensional polymeric structure extending parallel to (102). In the crystal, the nearest Ag⋯Ag distance between polymeric sheets is 3.8122 (12) Å. The crystal studied was a twin with a contribution of 0.2108 (12) for the minor component.

## Abstract 2

Cyanide anions bridge Fe^II^ and Ag^I^ cations to form a two-dimensional polymeric compound.

## Chemical context   

Metal–organic frameworks (MOFs), also known as porous coordination polymers, form a group of compounds that consist of metal ions and organic ligand linkers (Zhou & Kitagawa, 2014 ▸). MOFs have attracted considerable attention over the past decades due to the ability to tune their porosity, structure and other properties by a rational choice of the metal and linkers. Despite the fact that the most investigated properties of MOFs are gas storage and separation, it has been shown that the incorporation of corresponding building blocks or guests into MOFs can provoke specific functional magnetic, chiral, catalytic, conductive, luminescence and other properties.
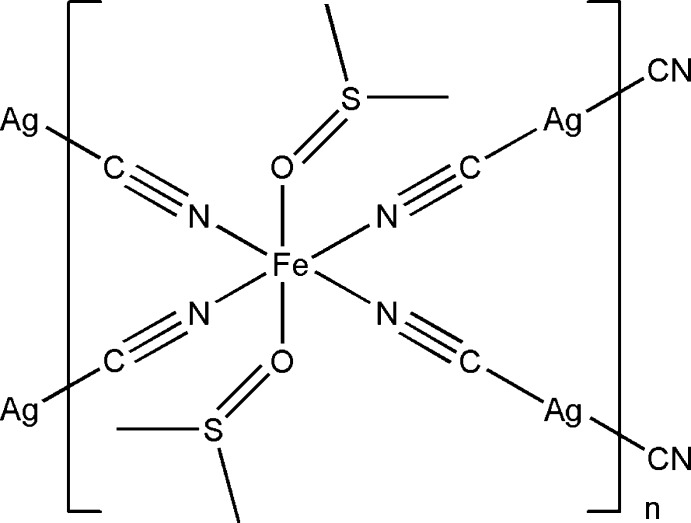


Hofmann clathrate analogues represent a huge group of MOFs. The first prototype clathrate of this family was [Ni(NH_3_)_2_{Ni(CN)_4_}] reported by Hofmann & Küspert (1897 ▸), however its structure was only obtained by Powell & Rayner (1949 ▸). The structure analysis showed that the coordination framework of this complex is supported by bridging square-planar tetracyanidonickelate ligands, and the octahedral coordination sphere of Ni^II^ is completed by two NH_3_ mol­ecules. The layers in this clathrate are separated by ∼8 Å, which leads to the formation of guest-accessible cavities. This has allowed a series of clathrates to obtained with different aromatic guests such as benzene, phenol, aniline, pyridine, thio­phene and pyrrole. Later, the group of Hofmann clathrate analogues was expanded to [*M*(*L*)_2_{*M*′(CN)_4_}] where *M* = Fe^2+^, Co^2+^, Ni^2+^, Cu^2+^, Zn^2+^, Cd^2+^ and Mn^2+^, *M*′ = Ni^2+^, Pd^2+^, Pt^2+^ and *L* is either a unidentate or bridging ligand to form two- or three-dimensional coordination frameworks, respectively.

More importantly, due to the rational choice of ligand, Kitazawa *et al.* (1996 ▸) succeeded in obtaining the first Hofmann-type complex [Fe(py)_2_{Ni(CN)_4_}] that exhibited spin-crossover behavior. This phenomenon is a spectacular ability of some 3*d* metals to exist in two different spin states. This discovery has led to multiple attempts to modify this compound in order to obtain other spin-crossover materials. The main synthetic approaches are: (*a*) the change of the pyridine ligand to other unidentate or bridging ligands; (*b*) the induction of various guest mol­ecules that influence spin-crossover characteristics; (*c*) use of different square-planar {[*M*(CN)_4_]^2−^, *M* = Ni^2+^, Pt^2+^, Pd^2+^; Kucheriv *et al.*, 2016 ▸}, dodeca­hedral {[Nb(CN)_8_]^4−^; Ohkoshi *et al.*, 2013 ▸} or linear {[*M*(CN)_2_]^−^, *M* = Ag^+^, Au^+^; Gural’skiy *et al.*, 2016*b* ▸} linkers. Here we offer a new Hofmann-like coordination compound with general formula [Fe(dmso)_2_{Ag(CN)_2_}_2_] in which the Fe^II^ atoms are stabilized in a high-spin state.

## Structural commentary   

**Figure 1:**
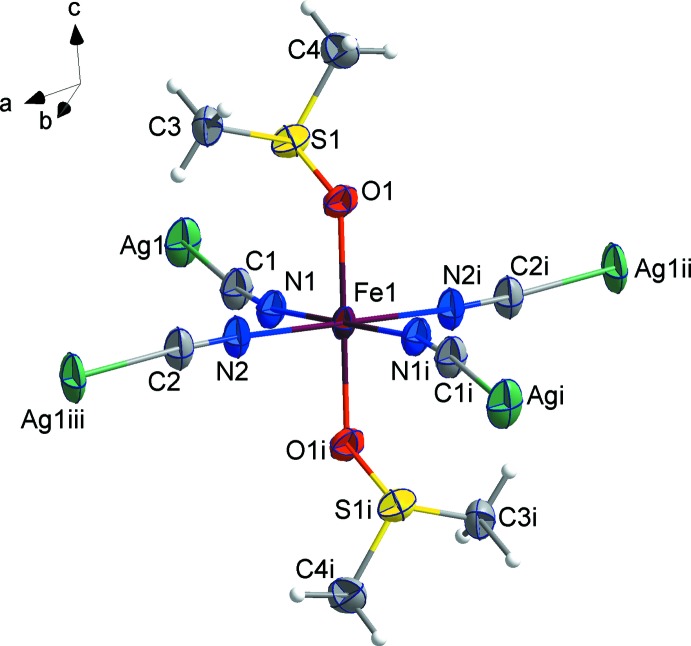
coordination environments of the Fe^II^ and Ag^I^ atoms in the structure of the title compound, showing the atom-labelling scheme, with displacement ellipsoids are drawn at the 50% probability level. [Symmetry codes: (i) 2 − *x*, 1 − *y*, 1 − *z*; (ii) 1 + *x*, 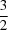 − *y*, 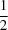 + *z*; (iii) 1 − *x*, −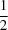 + *y*, 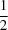 − *z*.]

The crystal structure of the title compound was determined from 243 K data. The Fe^II^ cation is located at an inversion centre and coordinated by four CN^−^ anions and two di­methyl­sulfoxide mol­ecules in a slightly compressed N_4_O_2_ octa­hedral environment (Fig. 1 ▸). The Ag^I^ cation is C-coord­inated by two CN^−^ anions in a nearly linear mode [C1—Ag—C2 = 173.0 (3)°]. The CN^−^ anions bridge the Fe^II^ and Ag^I^ cations to form a two-dimensional polymeric structure. In the structure, the equatorial Fe—N bonds [2.166 (4) and 2.176 (4) Å] have the typical value for Fe^II^ in a high-spin state. The axial positions of the Fe^II^ cation are occupied by two di­methyl­sulfoxide mol­ecules with an Fe—O bond length of 2.096 (4) Å. The S=O bond length of 1.532 (4) Å is increased by 0.03 Å with respect to non-coord­inating dmso; the average S—C bond of 1.774 (6) Å is shorter than in those in non-coordinating di­methyl­sulfoxide. This is a typical value for O-bonded di­methyl­sulfoxide complexes (Calligaris, 2004 ▸). The torsion angles around the Fe—O bond are Fe1—O1—S1—C3 = 96.3 (3)° and Fe1—O1—S1—C4 = −159.2 (3)°. The polyhedral distortion which is described by the deviation from an octa­hedral geometry is ΣFe|90 − Θ| = 9.86 (16)° where Θ is the N—Fe—N or O—Fe—N angle in the coordination environment of the metal; however, this value is slightly lower than expected for a high-spin Fe^II^ complex.

## Supra­molecular features   

**Figure 2:**
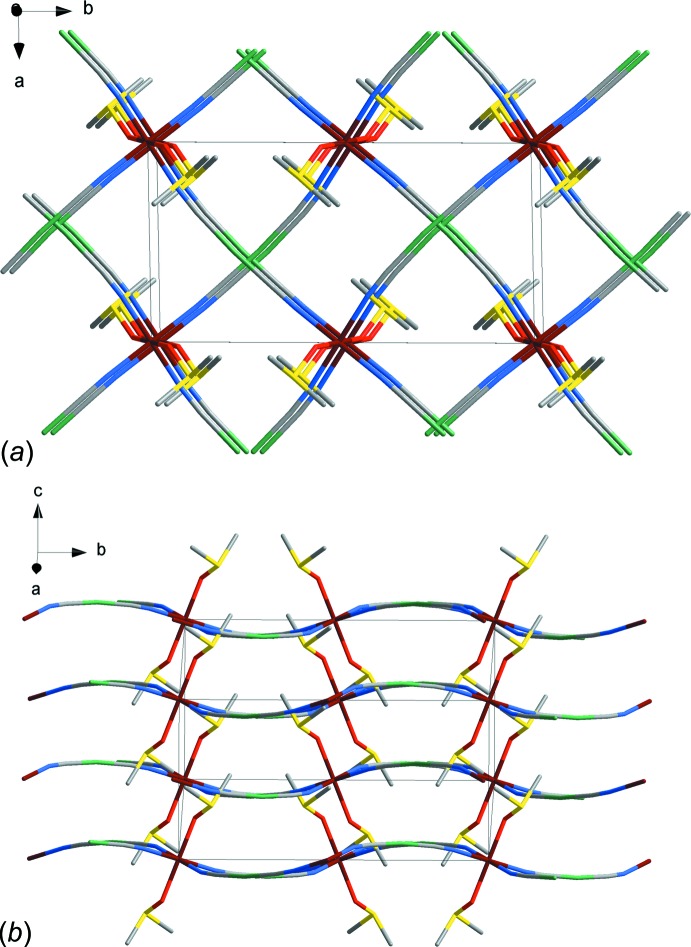
(a) View of the crystal structure of the title compound in the *ab* plane. H atoms have been omitted for clarity. (b) View of the crystal structure showing the two-dimensional layers. Colour key: brown Fe, green Ag, yellow S, blue N, grey C and red O.

The coordination framework is connected by bridging di­cyanido­argentate moieties into a two-dimensional grid that propagates along the (102) plane (Fig. 2 ▸ *a*). The short inter­layer Ag⋯Ag distance of 3.8122 (12) Å indicates argentophilic inter­actions that propagate along the *c-*axis direction. A similar type of inter­molecular bonding between seemingly closed-shell metal atoms has previously been reported for many Ag- and Au-containing Hofmann-type structures, *e.g.* Au⋯Au distances of 3.3792 (3) Å were found between the [Fe{Au(CN)_2_}^−^] planes (Gural’skiy *et al.*, 2016*a* ▸). In addition, in the title compound the Fe—N—C and Ag—C—N linkages show a slight deviation from linearity (9.5 and 6° on average, respectively) that leads to a slight corrugation of [Fe{Ag(CN)_2_}^−^] layers (Fig. 2 ▸ *b*).

## Database survey   

The title compound has never been obtained before. A database survey reveals numerous Fe–Ag CN-bridged frameworks supported by various co-ligands axially bound to the iron atoms.

## Synthesis and crystallization   

Crystals of the title compound were obtained by the slow-diffusion method within three layers in 10 ml tubes: the first layer was a solution of Fe(ClO_4_)_2_ (0.1 mmol, 26 mg) in di­methyl­sulfoxide (2 ml); second one was a di­methyl­sulfoxide–ethanol mixture (1:1, 5 ml); the third was a solution of K[Ag(CN)_2_] (0.1 mmol, 20 mg) in an ethanol–water mixture (9:1 ratio *v*/*v*, 2 ml). After two weeks, orange crystals grew in the second layer; they were collected and kept under the mother solution prior to the measurements.

## Refinement   

Crystal data, data collection and structure refinement details are summarized in Table 1 ▸. All H atoms of methyl groups were placed geometrically at their expected calculated positions with C—H = 0.97 Å and *U*_iso_(H) = 1.5*U*_eq_(C). The idealized CH_3_ group was fixed using an AFIX 137 command that allowed the H atoms to ride on C atom and rotate around S—C bond. Twining of two components was considered with the transformation matrix (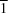 0 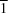 0 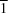 0 0 0 1) and a twin contribution of BASF = 0.2108 (12).

## Supplementary Material

Crystal structure: contains datablock(s) global, I. DOI: 10.1107/S2056989017001049/xu5899sup1.cif

Structure factors: contains datablock(s) I. DOI: 10.1107/S2056989017001049/xu5899Isup2.hkl

CCDC reference: 1528660

Additional supporting information:  crystallographic information; 3D view; checkCIF report

## Figures and Tables

**Table 1:** Experimental details

Crystal data	
Chemical formula	[Ag_2_Fe(CN)_4_(C_2_H_6_OS)_2_]
*M*_r_	531.93
Crystal system, space group	Monoclinic, *P*2_1_/*c*
Temperature (K)	243
*a*, *b*, *c* (Å)	8.4125 (16), 14.492 (3), 7.4679 (14)
β (°)	116.053 (4)
*V* (Å^3^)	817.9 (3)
*Z*	2
Radiation type	Mo *K*α
μ (mm^−1^)	3.50
Crystal size (mm)	0.15 × 0.1 × 0.05

Data collection	
Diffractometer	Bruker SMART
Absorption correction	Multi-scan (*SADABS*; Bruker, 2013 ▸)
*T*_min_, *T*_max_	0.625, 0.746
No. of measured, independent and observed [*I* > 2σ(*I*)] reflections	16468, 1970, 1726
*R*_int_	0.045
(sin θ/λ)_max_ (Å^−1^)	0.661

Refinement	
*R*[*F* ^2^ > 2σ(*F* ^2^)], *wR*(*F* ^2^), *S*	0.031, 0.071, 1.16
No. of reflections	1970
No. of parameters	91
H-atom treatment	H-atom parameters constrained
Δρ_max_, Δρ_min_ (e Å^−3^)	0.42, −1.05

Computer programs: *SMART* and *SAINT* (Bruker, 2013 ▸), *SHELXS97* (Sheldrick, 2008 ▸), *SHELXL2014* (Sheldrick, 2015 ▸), *DIAMOND* (Brandenburg *et al.*, 1999 ▸) and *publCIF* (Westrip, 2010 ▸).

## supplementary crystallographic information

### Crystal data

**Table:**

[Ag_2_Fe(CN)_4_(C_2_H_6_OS)_2_]	*F*(000) = 512
*M**_r_* = 531.93	*D*_x_ = 2.160 Mg m^−^^3^
Monoclinic, *P*2_1_/*c*	Mo *K*α radiation, λ = 0.71073 Å
*a* = 8.4125 (16) Å	Cell parameters from 4840 reflections
*b* = 14.492 (3) Å	θ = 2.7–26.2°
*c* = 7.4679 (14) Å	µ = 3.50 mm^−^^1^
β = 116.053 (4)°	*T* = 243 K
*V* = 817.9 (3) Å^3^	Plate, orange
*Z* = 2	0.15 × 0.1 × 0.05 mm

### Data collection

**Table:**

Bruker SMART diffractometer	1726 reflections with *I* > 2σ(*I*)
φ and ω scans	*R*_int_ = 0.045
Absorption correction: multi-scan (SADABS; Bruker, 2013)	θ_max_ = 28.0°, θ_min_ = 1.4°
*T*_min_ = 0.625, *T*_max_ = 0.746	*h* = −11→11
16468 measured reflections	*k* = −19→19
1970 independent reflections	*l* = −8→9

### Refinement

**Table:**

Refinement on *F*^2^	Primary atom site location: structure-invariant direct methods
Least-squares matrix: full	Hydrogen site location: inferred from neighbouring sites
*R*[*F*^2^ > 2σ(*F*^2^)] = 0.031	H-atom parameters constrained
*wR*(*F*^2^) = 0.071	*w* = 1/[σ^2^(*F*_o_^2^) + (0.0068*P*)^2^ + 2.779*P*] where *P* = (*F*_o_^2^ + 2*F*_c_^2^)/3
*S* = 1.16	(Δ/σ)_max_ = 0.001
1970 reflections	Δρ_max_ = 0.42 e Å^−^^3^
91 parameters	Δρ_min_ = −1.05 e Å^−^^3^
0 restraints	

### Special details

**Table:**

Geometry. All esds (except the esd in the dihedral angle between two l.s. planes) are estimated using the full covariance matrix. The cell esds are taken into account individually in the estimation of esds in distances, angles and torsion angles; correlations between esds in cell parameters are only used when they are defined by crystal symmetry. An approximate (isotropic) treatment of cell esds is used for estimating esds involving l.s. planes.

### Fractional atomic coordinates and isotropic or equivalent isotropic displacement parameters (Å^2^)

**Table:**

	*x*	*y*	*z*	*U*_iso_*/*U*_eq_	
Ag1	0.45426 (6)	0.72349 (3)	0.33300 (9)	0.05178 (15)	
Fe1	1.0000	0.5000	0.5000	0.0255 (2)	
S1	1.17380 (18)	0.39095 (9)	0.9253 (2)	0.0377 (3)	
C3	1.3090 (7)	0.4721 (4)	1.1049 (9)	0.0494 (16)	
H3A	1.3567	0.5158	1.0427	0.074*	
H3B	1.4054	0.4402	1.2119	0.074*	
H3C	1.2389	0.5049	1.1586	0.074*	
C4	1.0778 (8)	0.3340 (4)	1.0651 (9)	0.0478 (15)	
H4A	1.0194	0.3789	1.1121	0.072*	
H4B	1.1697	0.3029	1.1782	0.072*	
H4C	0.9920	0.2890	0.9811	0.072*	
O1	1.0225 (5)	0.4471 (3)	0.7713 (5)	0.0376 (8)	
N1	0.7675 (5)	0.5774 (3)	0.4638 (7)	0.0349 (10)	
C1	0.6498 (6)	0.6262 (4)	0.4204 (9)	0.0404 (12)	
N2	0.1741 (5)	0.8859 (3)	0.1555 (7)	0.0391 (11)	
C2	0.2720 (7)	0.8283 (4)	0.2252 (10)	0.0419 (13)	

### Atomic displacement parameters (Å^2^)

**Table:**

	*U*^11^	*U*^22^	*U*^33^	*U*^12^	*U*^13^	*U*^23^
Ag1	0.03045 (19)	0.0337 (2)	0.0765 (3)	0.01486 (16)	0.0099 (2)	0.0063 (2)
Fe1	0.0182 (4)	0.0186 (4)	0.0328 (5)	0.0011 (3)	0.0048 (4)	0.0001 (4)
S1	0.0387 (7)	0.0344 (6)	0.0375 (7)	0.0129 (5)	0.0146 (6)	0.0053 (5)
C3	0.029 (3)	0.057 (4)	0.053 (4)	−0.006 (2)	0.010 (3)	0.008 (3)
C4	0.057 (4)	0.035 (3)	0.045 (3)	−0.007 (3)	0.017 (3)	0.002 (3)
O1	0.0322 (18)	0.041 (2)	0.034 (2)	0.0086 (16)	0.0097 (16)	0.0076 (17)
N1	0.0260 (19)	0.033 (2)	0.039 (3)	0.0043 (16)	0.0084 (18)	−0.0005 (19)
C1	0.028 (2)	0.035 (3)	0.051 (3)	0.006 (2)	0.011 (2)	0.001 (3)
N2	0.025 (2)	0.027 (2)	0.054 (3)	0.0036 (17)	0.007 (2)	0.001 (2)
C2	0.028 (2)	0.031 (3)	0.057 (4)	0.003 (2)	0.009 (3)	0.000 (3)

### Geometric parameters (Å, º)

**Table:**

Ag1—C1	2.044 (5)	S1—O1	1.523 (4)
Ag1—C2	2.054 (5)	C3—H3A	0.9700
Fe1—O1^i^	2.096 (4)	C3—H3B	0.9700
Fe1—O1	2.096 (4)	C3—H3C	0.9700
Fe1—N1	2.166 (4)	C4—H4A	0.9700
Fe1—N1^i^	2.166 (4)	C4—H4B	0.9700
Fe1—N2^ii^	2.176 (4)	C4—H4C	0.9700
Fe1—N2^iii^	2.176 (4)	N1—C1	1.142 (6)
S1—C3	1.771 (6)	N2—Fe1^iv^	2.176 (4)
S1—C4	1.778 (6)	N2—C2	1.127 (6)
			
C1—Ag1—C2	173.0 (3)	O1—S1—C4	104.3 (3)
O1^i^—Fe1—O1	180.0	S1—C3—H3A	109.5
O1—Fe1—N1^i^	89.82 (16)	S1—C3—H3B	109.5
O1—Fe1—N1	90.18 (16)	S1—C3—H3C	109.5
O1^i^—Fe1—N1	89.82 (16)	H3A—C3—H3B	109.5
O1^i^—Fe1—N1^i^	90.18 (16)	H3A—C3—H3C	109.5
O1^i^—Fe1—N2^ii^	89.54 (17)	H3B—C3—H3C	109.5
O1—Fe1—N2^iii^	89.54 (17)	S1—C4—H4A	109.5
O1^i^—Fe1—N2^iii^	90.46 (17)	S1—C4—H4B	109.5
O1—Fe1—N2^ii^	90.46 (17)	S1—C4—H4C	109.5
N1—Fe1—N1^i^	180.0	H4A—C4—H4B	109.5
N1^i^—Fe1—N2^iii^	91.82 (16)	H4A—C4—H4C	109.5
N1^i^—Fe1—N2^ii^	88.18 (16)	H4B—C4—H4C	109.5
N1—Fe1—N2^ii^	91.83 (16)	S1—O1—Fe1	128.0 (2)
N1—Fe1—N2^iii^	88.17 (16)	C1—N1—Fe1	168.3 (5)
N2^ii^—Fe1—N2^iii^	180.0	N1—C1—Ag1	173.7 (5)
C3—S1—C4	99.8 (3)	C2—N2—Fe1^iv^	173.6 (5)
O1—S1—C3	105.2 (3)	N2—C2—Ag1	175.5 (6)
			
C3—S1—O1—Fe1	96.3 (3)	C4—S1—O1—Fe1	−159.2 (3)

Symmetry codes: (i) −*x*+2, −*y*+1, −*z*+1; (ii) *x*+1, −*y*+3/2, *z*+1/2; (iii) −*x*+1, *y*−1/2, −*z*+1/2; (iv) −*x*+1, *y*+1/2, −*z*+1/2.
